# Oxygen-Induced and pH-Induced Direct Current Artifacts on Invasive Platinum/Iridium Electrodes for Electrocorticography

**DOI:** 10.1007/s12028-021-01358-2

**Published:** 2021-10-07

**Authors:** Sebastian Major, Nenad Gajovic-Eichelmann, Johannes Woitzik, Jens P. Dreier

**Affiliations:** 1 Center for Stroke Research Berlin, Campus Charité Mitte, Charité–Universitätsmedizin Berlin, corporate member of Freie Universität Berlin, Humboldt-Universität zu Berlin, and Berlin Institute of Health, Charitéplatz 1, 10117 Berlin, Germany; 2 Department of Experimental Neurology, Charité–Universitätsmedizin Berlin, corporate member of Freie Universität Berlin, Humboldt-Universität zu Berlin, and Berlin Institute of Health, Berlin, Germany; 3 Department of Neurology, Charité–Universitätsmedizin Berlin, corporate member of Freie Universität Berlin, Humboldt-Universität zu Berlin, and Berlin Institute of Health, Berlin, Germany; 4 Fraunhofer Institute for Cell Therapy and Immunology–Branch Bioanalytics and Bioprocesses, Potsdam, Germany; 5 Department of Neurosurgery, Evangelisches Krankenhaus Oldenburg, University of Oldenburg, Oldenburg, Germany; 6 Bernstein Center for Computational Neuroscience Berlin, Berlin, Germany; 7 Einstein Center for Neurosciences Berlin, Berlin, Germany

**Keywords:** Multimodal monitoring, Cytotoxic edema, Spreading depolarization, Electrocorticography, Aneurysmal subarachnoid hemorrhage, Traumatic brain injury, Platinum electrode

## Abstract

**Background:**

Spreading depolarization (SD) and the initial, still reversible phase of neuronal cytotoxic edema in the cerebral gray matter are two modalities of the same process. SD may thus serve as a real-time mechanistic biomarker for impending parenchyma damage in patients during neurocritical care. Using subdural platinum/iridium (Pt/Ir) electrodes, SD is observed as a large negative direct current (DC) shift. Besides SD, there are other causes of DC shifts that are not to be confused with SD. Here, we systematically analyzed DC artifacts in ventilated patients by observing changes in the fraction of inspired oxygen. For the same change in blood oxygenation, we found that negative and positive DC shifts can simultaneously occur at adjacent Pt/Ir electrodes.

**Methods:**

Nurses and intensivists typically increase blood oxygenation by increasing the fraction of inspired oxygen at the ventilator before performing manipulations on the patient. We retrospectively identified 20 such episodes in six patients via tissue partial pressure of oxygen (p_ti_O_2_) measurements with an intracortical O_2_ sensor and analyzed the associated DC shifts. In vitro, we compared Pt/Ir with silver/silver chloride (Ag/AgCl) to assess DC responses to changes in pO_2_, pH, or 5-min square voltage pulses and investigated the effect of electrode polarization on pO_2_-induced DC artifacts.

**Results:**

Hyperoxygenation episodes started from a p_ti_O_2_ of 37 (30–40) mmHg (median and interquartile range) reaching 71 (50–97) mmHg. During a total of 20 episodes on each of six subdural Pt/Ir electrodes in six patients, we observed 95 predominantly negative responses in six patients, 25 predominantly positive responses in four patients, and no brain activity changes. Adjacent electrodes could show positive and negative responses simultaneously. In vitro, Pt/Ir in contrast with Ag/AgCl responded to changes in either pO_2_ or pH with large DC shifts. In response to square voltage pulses, Pt/Ir falsely showed smaller DC shifts than Ag/AgCl, with the worst performance under anoxia. In response to pO_2_ increase, Pt/Ir showed DC positivity when positively polarized and DC negativity when negatively polarized.

**Conclusions:**

The magnitude of pO_2_-induced subdural DC shifts by approximately 6 mV was similar to that of SDs, but they did not show a sequential onset at adjacent recording sites, could be either predominantly negative or positive in contrast with the always negative DC shifts of SD, and were not accompanied by brain activity depression. Opposing polarities of pO_2_-induced DC artifacts may result from differences in baseline electrode polarization or subdural p_ti_O_2_ inhomogeneities relative to subdermal p_ti_O_2_ at the quasi-reference.

**Supplementary Information:**

The online version contains supplementary material available at 10.1007/s12028-021-01358-2.

## Introduction

Spreading depolarization (SD) and the initial, still reversible phase of neuronal cytotoxic edema in the cerebral gray matter are two modalities of the same process [1]. The term SD describes the electrophysiological aspect, and the term cytotoxic edema describes the pathomorphological aspect of this process. Accordingly, SD is observed as a large negative direct current (DC) shift, two-photon microscopy shows neuronal swelling with a beaded morphology of neuronal dendrites during SD [2], and magnetic resonance imaging shows a propagating wave of diffusion restriction [3–5]. SDs occur during a plethora of clinical conditions, including migraine aura [6, 7], ischemic stroke [8, 9], traumatic brain injury [10, 11], aneurysmal subarachnoid hemorrhage (aSAH) and delayed ischemic stroke [12–14], spontaneous intracerebral hemorrhage [15], subdural hematoma [16], development of brain death [17, 18], and dying from circulatory arrest [19]. The SD continuum describes the spectrum from transient events of intermediate duration to short duration in less ischemic or adequately supplied tissue to terminal SDs in severely ischemic tissue characterized by the transition of the neurons from the state of injury to cell death [20, 21]. Dying is characterized by the transition from this negative DC shift to the so-called negative ultraslow potential in electrophysiology and continued diffusion restriction in diffusion-weighted magnetic resonance imaging [12, 17, 18, 22–24]. Thus, terminal SD is composed of the initial SD phase followed by the SD-initiated negative ultraslow potential phase [12].

In addition to the assessment of SD and neuronal cytotoxic edema, electrocorticography (ECoG) also allows the assessment of spontaneous brain activity in the neural network. Spontaneous brain activity results from the firing of neurons. The firing of upstream neurons leads to postsynaptic potentials in downstream neurons. These cause rapid extracellular field potential changes that can be recorded in the alternating current (AC) frequency range greater than 0.5 Hz, as most cortical neurons have a similar spatial orientation so that the many potential changes of individual neurons add up sufficiently. SDs can cooccur with two different types of depression of this spontaneous activity: nonspreading depression and spreading depression [24, 25]. In less ischemic or adequately supplied tissue with spontaneous activity, SD induces spreading depression of that activity. In contrast, neuronal hyperpolarization and nonspreading depression precede SD in severely ischemic brain regions so that SD occurs, but it cannot induce spreading depression in those regions. Only when SD reaches electrically active tissue during its migration can it then induce spreading depression. Importantly, SDs are fundamentally different from epileptic seizures and can be easily and unequivocally distinguished from them by ECoG [26]. Although SDs may cooccur with electrographic seizures during status epilepticus [27, 28], the type of excitability increase that leads to epileptic seizures is sometimes diametrically different from the type of excitability increase that leads to SDs [29–33]. Based on all these observations, it has been suggested that SD may serve as a real-time mechanistic biomarker for impending parenchyma damage in patients during neurocritical care [24, 34].

During neurocritical care, SDs are recorded subdurally with platinum/iridium (Pt/Ir) disposable electrodes, which are also used in the presurgical diagnostics of patients with epilepsy [35, 36]. This material is less suitable for recording DC potential shifts than silver/silver chloride (Ag/AgCl), the gold standard in experimental electrophysiology, but Ag/AgCl is rightly banned for subdural use in humans because of neurotoxic side effects. Unfavorable properties of Pt electrodes are their polarizability and much higher impedance, which may exceed 100 kiloohm with small DC charging currents [37]. Alternatively, stainless steel can be clinically used for subdural ECoG. Stainless steel is relatively inexpensive but also polarizable, and its low-frequency noise is significantly higher than that of platinum [35, 37, 38]. Low-frequency noise stands for variations in the rate of drift in DC recordings due to slow spontaneous changes in electrode polarization. Thus, thin-film Pt electrodes are currently the best available option for the monitoring of SDs in humans [36, 39, 40].

**Fig. 1:**
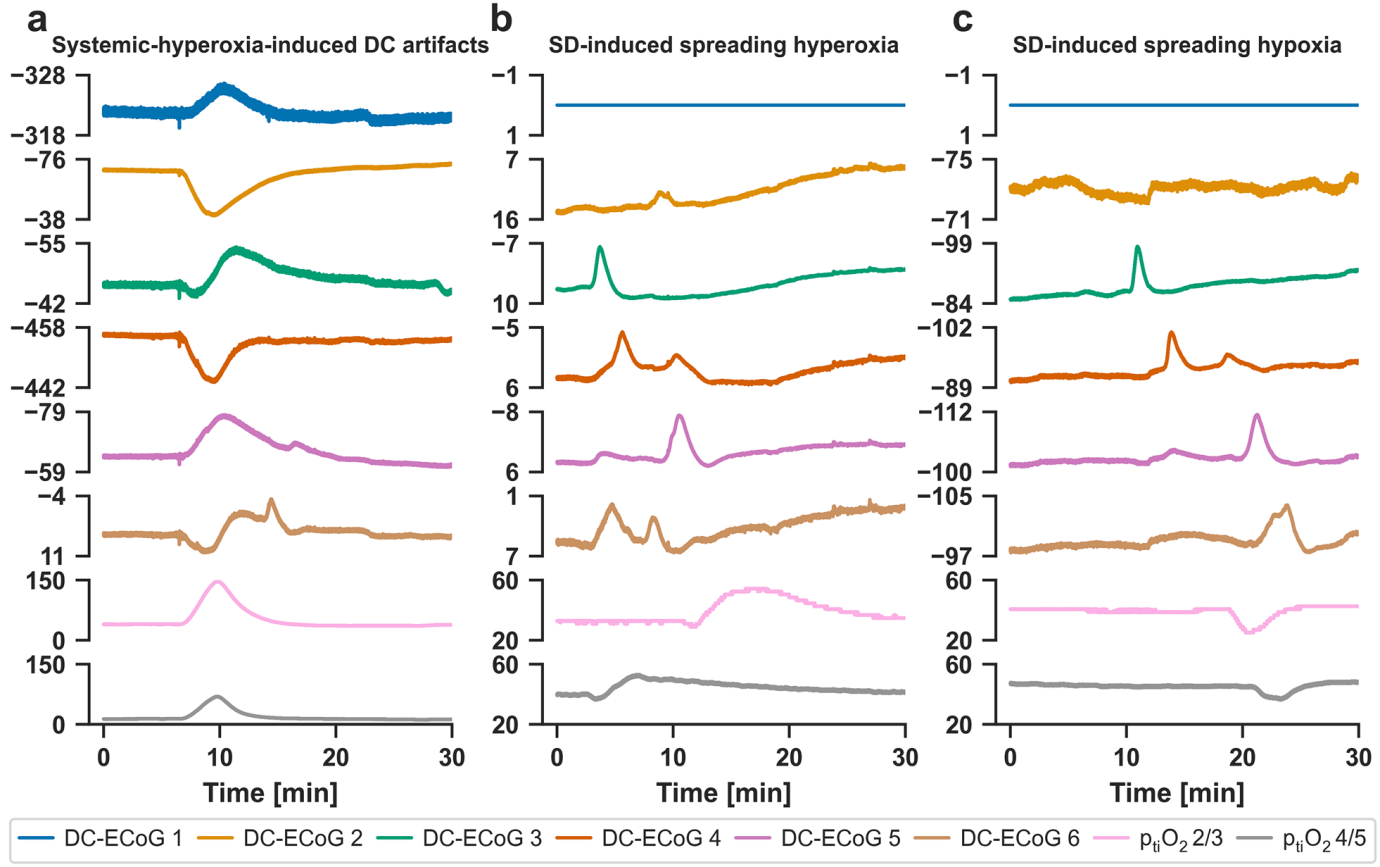
Systemic hyperoxygenation-induced DC shifts in **a** subdural DC-ECoG recordings versus, **b** SD-induced spreading hyperoxia and **c** SD-induced spreading hypoxia in the brain cortex after aSAH. Traces 1–6 from top to bottom give the DC-ECoG recordings from electrodes 1–6 in mV (6-contact Wyler recording strip, platinum [Pt] contacts with 4.2-mm^2^ exposed surface spaced at 10 mm along the strip). Brain p_ti_O_2_ in mmHg was measured here in the cerebral cortex using two O_2_ sensors between electrodes 2 and 3 (trace 7) and electrodes 4 and 5 (trace 8). Parts **a** and **b/c** come from two different patients. **a** Nurses and intensivists can modify blood oxygenation by increasing the FiO_2_ at the ventilator. This increases blood oxygenation diffusely throughout the body and thus also increases the partial pressure of O_2_ in the subdural space. The increase in O_2_ tension in the subdural space typically results in high-amplitude DC shifts at the subdural electrodes, which, interestingly, can be positive or negative at adjacent electrodes. To the human eye, these O_2_-induced DC artifacts in subdural DC-ECoG recordings are relatively easy to distinguish from SDs because the dominant DC component of SDs is always negative and SDs typically propagate between adjacent recording sites. However, the hyperoxia-induced DC shifts could cause problems for a simple automatic detection algorithm based only on DC shifts, or if an investigator is not aware that changes in blood oxygenation can cause such DC artifacts, as they could then be mistaken for SDs. It should be noted that as FiO_2_ increases, O_2_ tension increases, not only in the subdural space, but everywhere and in the brain tissue. In the intracortical p_ti_O_2_ measurements, it is remarkable how differently the same change in blood oxygenation quantitatively affects the two O_2_ sensors, which are only about 1 cm apart and should be at the same cortical depth. Theoretically, there could be technical reasons for this discrepancy concerning the probes [68]. In the area of tissue probes, there is also typically an inflammatory reaction with opening of the blood–brain barrier [63]. Vasogenic edema could increase the distance of the sensor to the nearest capillary bed. Depending on the severity of the vasogenic edema around different probes, this could affect the measurements differently. It could also be that capillary perfusion is locally inhomogeneous at the two sensors as a result of pathological changes in vascular tone after aSAH [69]. But even for healthy brain tissue, a so-called oxygen field is typical and shows large local gradients due to local tissue respiration [70]. Homogeneous O_2_ values are therefore not to be expected even in healthy tissue. **b**, **c** SDs typically lead to pronounced changes in p_ti_O_2_ in brain tissue [14, 43, 44, 50, 71, 72]. Part **b** shows an SD-induced spreading hyperoxia that was measured in a patient with aSAH 10 h before the SD-induced spreading hypoxia displayed in **c**. Such a change of SD-induced p_ti_O_2_ responses from spreading hyperoxia to spreading hypoxia over time could occur after aSAH as a result of the shift from normal to inverse hemodynamic responses to SD [14, 24, 44, 51]. However, in the present case, the hypoxic oxygen response starts from a higher baseline level than the hyperoxic oxygen response, the lowest p_ti_O_2_ level during SD is the same in both SDs, and the negative DC shifts have approximately the same duration in both SDs. Thus, although the SD in **c** induces spreading hypoxia, the response does not meet the criteria that would be indicative of spreading ischemia [24]. Importantly, although SDs cause such pronounced p_ti_O_2_ responses in brain tissue, they are very unlikely to cause comparable p_ti_O_2_ responses in the subdural space because, first, SD is a phenomenon of the brain’s gray matter and not a phenomenon of the meninges and, second, the O_2_ tension of the subdural space depends on perfusion of the capillaries of the meninges and not the capillaries of the brain. Consistent with this, in **b** and **c**, whether p_ti_O_2_ increases or decreases in the brain tissue during SD does not affect the polarity of DC changes measured with Pt electrodes in the subdural space during SD. AC, alternating current, aSAH, aneurysmal subarachnoid hemorrhage, DC, direct current, ECoG, electrocorticography, FiO_2_, fraction of inspired O_2_, p_ti_O_2_, tissue partial pressure of oxygen, SD, spreading depolarization

**Fig. 2:**
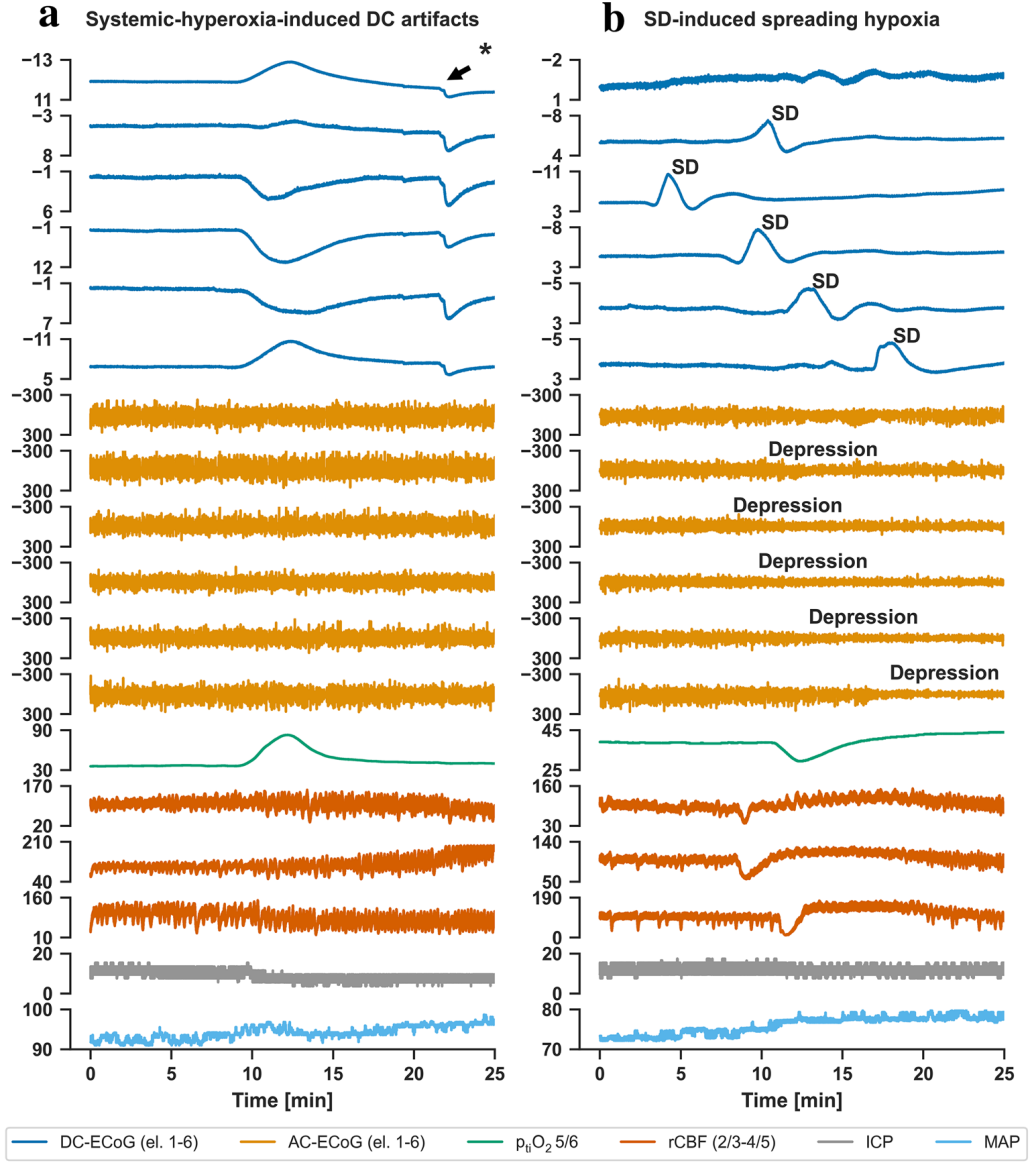
Multimodal recordings of **a** systemic hyperoxygenation-induced DC artifacts versus **b** SD-induced spreading hypoxia in the same patient with aSAH. Traces 1–6 from top to bottom give the DC-ECoG recordings from electrodes 1–6 in mV (* represents movement artifact). Traces 7–12 show the bandpass filtered AC-ECoG of the same electrodes in µV (frequency band between 0.5 and 45 Hz). Trace 13 shows p_ti_O_2_ recorded in mmHg with an intracortical sensor between electrodes 5 and 6. Traces 14–16 show rCBF measured with optodes between electrodes 2 and 3, 3 and 4, and 4 and 5 from top to bottom by laser Doppler flowmetry. Trace 17 provides the intracranial pressure in mmHg measured using an extraventricular drainage catheter and trace 18 the mean arterial pressure in mmHg measured using a catheter in the radial artery. In addition to the fact that the dominant DC component of SDs is always negative and SDs typically propagate between adjacent recording sites, there are other criteria that help to distinguish SDs from O_2_-induced DC artifacts. These include, for example, that SD typically induces spreading depression of spontaneous activity in tissue with spontaneous electrical activity. SD-induced spreading depression is observed in the AC-ECoG recordings as a rapidly developing reduction in the amplitudes of spontaneous activity which spreads together with SD between adjacent recording sites. In addition, SD induces characteristic rCBF changes. In healthy tissue, an increase in rCBF typically dominates initially, which is called spreading hyperemia, and is followed by a mild decrease in blood flow called oligemia [73]. In contrast, under pathological conditions, instead of an initial vasodilation, SD can induce a severe initial vasoconstriction (equals inverse hemodynamic response), which can be long-lasting and lead to impaired neuronal repolarization with prolongation of SD as a consequence of an insufficient supply with oxidative substrates [51]. This phenomenon is called spreading ischemia and may lead to infarction after a certain duration both in animals and humans [12, 74]. The hemodynamic responses to SD typically show a continuum across tissue from an inverse ischemic response to an increasingly normal hyperemic response or the other way round. In the present case, we see that SD induces inverse rCBF responses at all three optodes, but these are not yet of precarious proportions, because there is a relatively rapid recovery of rCBF. The initial drop in rCBF is then followed by a transient hyperemia which is typical of such inverse responses. AC, alternating current, aSAH, aneurysmal subarachnoid hemorrhage, DC, direct current, ECoG, electrocorticography, MAP, mean arterial pressure, p_ti_O_2_, tissue partial pressure of oxygen, rCBF, regional cerebral blood flow, SD, spreading depolarization

However, unfortunately, not all large DC shifts recorded in patients are SDs. Besides movement artifacts, there is at least one other type of DC artifact that is significantly more complex and specifically related to Pt electrodes. Thus, it was previously noted in two clinical cases that the interference of tissue partial pressure of oxygen (p_ti_O_2_) with subdural platinum electrodes produces DC artifacts [19, 41]. These artifacts are particularly impressive when nurses or intensivists increase blood oxygenation by increasing the fraction of inspired oxygen (FiO_2_) on the ventilator (Figs. 1a, 2a). In the present article, we systematically investigated these artifacts and compared them with SDs. Patients are typically subjected to brief episodes of hyperoxygenation before manipulations are performed on them, especially manipulations around the endotracheal tube. Clearly, it is important for monitoring SDs to correctly detect these pO_2_-induced DC shifts as artifacts even in the absence of simultaneous measurements of p_ti_O_2_ with an intraparenchymal sensor. This is especially true because it is suspected that patient manipulations also simultaneously increase the risk for SDs [42]. A property of the pO_2_-induced DC artifacts that is surprising at first sight is that they can be simultaneously negative and positive in adjacent Pt electrodes. In further in vitro experiments, we took this as an opportunity to look more closely at pO_2_ and pH interferences of Pt electrodes to search for possible explanations of this peculiarity.

## Methods

### Patients

For the clinical study, we selected patients from a prospectively collected database using prespecified criteria, as described below. Table 1 provides demographic data. The protocol was approved by the ethics committee of the Charité – Universitätsmedizin Berlin. Either informed consent or surrogate informed consent was obtained for all patients, and research was conducted in accordance with the Declaration of Helsinki. Patients with aSAH consecutively enrolled in the Co-Operative Studies on Brain Injury Depolarizations (COSBID) at one center (Campus Virchow Klinikum, Berlin, Germany) were screened for study inclusion. Prospective inclusion criteria for COSBID have been described previously [43]. For this study, patients were further screened retrospectively for (1) the availability of DC/AC recordings (bandpass: 0–45 Hz) of sufficient quality, (2) p_ti_O_2_ recordings, and (3) at least one hyperoxygenation episode induced by an increase of FiO_2_ at the ventilator. Typically, FiO_2_ is briefly increased to 100% during such episodes. After the 6th consecutively screened patient with at least one hyperoxygenation episode, we analyzed the data. In total, 20 hyperoxygenation episodes could be analyzed.

**Table 1:** Baseline characteristic of the patients

No	Age (year)	Sex	WFNS grade	Modified Fisher scale [75]	Location of aneurysm	Treatment	Location of electrode
1	62	Female	4	3	ACoP-L	Clipping	Left frontal lobe
2	55	Male	5	4	ACoA	Clipping	Right frontal lobe
3	70	Female	4	4	ICA-L	Clipping	Left frontal lobe
4	79	Female	4	4	MCA-L	Clipping	Left frontal lobe
5	39	Male	4	4	PericA-L	Clipping	Right frontal lobe
6	54	Male	1	3	ACoA	Clipping	Right frontal lobe

*ACoA* anterior communicating artery, *ACoP* posterior communicating artery, *ICA* internal carotid artery, *L* left, *MCA* middle cerebral artery, *PericA* pericallosal artery, *WFNS* World Federation of Neurosurgical Societies

### Neuromonitoring

For the continuous DC/AC-ECoG recording, a six-contact, collinear Pt/Ir electrode strip (Wyler type, 5-mm diameter; 10-mm spacing; Ad-Tech Medical, Racine, WI) was placed on the cerebral cortex during aneurysm surgery as described previously [13, 14]. A subdermal Pt/Ir needle electrode at the forehead was used as a quasi-reference for monopolar (= unipolar = referential) recordings. Thus, the quasi-reference was well shielded from p_ti_O_2_, pH and potential changes generated by the brain, but it was affected by systemic hyperoxia. In the rest of the article, Pt/Ir electrodes are referred to simply as Pt electrodes. The full-band DC/AC-ECoG (0–45 Hz) was sampled at 200 Hz using a DC-coupled BrainAmp MR plus integrated amplifier and analog–digital converter and BrainVision Recorder software (Brain Products GmbH, Gilching, Germany). The p_ti_O_2_ was measured with a Clark-type intraparenchymal sensor (Licox CC1P1; Integra Lifesciences Corporation, Plainsboro, NJ) implanted below the brain surface next to the electrode strip as described previously [14, 43, 44]. In one of the patients with hyperoxygenation episodes, two O_2_ sensors were used. A subdural opto-electrode strip for the simultaneous measurement of DC/AC-ECoG and regional cerebral blood flow using laser Doppler flowmetry was placed in a subset of patients (Perimed AB, Järfälla, Sweden) [12, 14]. The four integrated optodes were connected to a Periflux 5000 (Perimed AB). Data were sampled at 200 Hz with a PowerLab 16/SP analog–digital converter and LabChart software (ADInstruments, New South Wales, Australia). Nurses and intensivists typically increase blood oxygenation by increasing FiO_2_ at the ventilator before performing manipulations on the patient. This often leads to movement artifacts. Hyperoxygenation episodes were included in the analysis only if no movement artifacts were present in the corresponding DC/AC-ECoG traces.

### In vitro experiments

Two chambers were filled with 5-mM phosphate buffer in 0.9% NaCl and connected with a salt bridge made of soaked filter paper. The same Pt electrodes as for patient recordings and an Ag/AgCl electrode were placed in the first chamber (= recording chamber) and connected to separate amplifier modules (LPBF-01GX; npi electronic GmbH, Tamm, Germany). Reference electrodes of the same materials as the two different types of recording electrodes were placed in the second chamber and attached to the corresponding amplifier modules. Accordingly, they could not be affected by pO_2_ and pH changes in the recording chamber. Amplifier modules were connected to a Power1401 mk II analog–digital converter (Cambridge Electronic Design Limited, Cambridge, UK). Copper (Cu) electrodes attached to the digital-analog converter output of the Power1401 were used for the external application of electric current to the experimental setup. A pH meter and O_2_-sensitive probe (Picoammeter PA 2000; Unisense, Aarhus, Denmark) were placed in the recording chamber. In contrast with the solution in the reference chamber, the solution in the recording chamber was continuously bubbled with a mixture of O_2_ and N_2_. The pO_2_ was set by adjusting the O_2_:N_2_ ratio until the desired value was reached while the total gas flow was kept constant (Supplemental Fig. 1). Changes in pH were achieved by addition of either NaOH or HCl and verified by the pH meter.

**Fig. 3:**
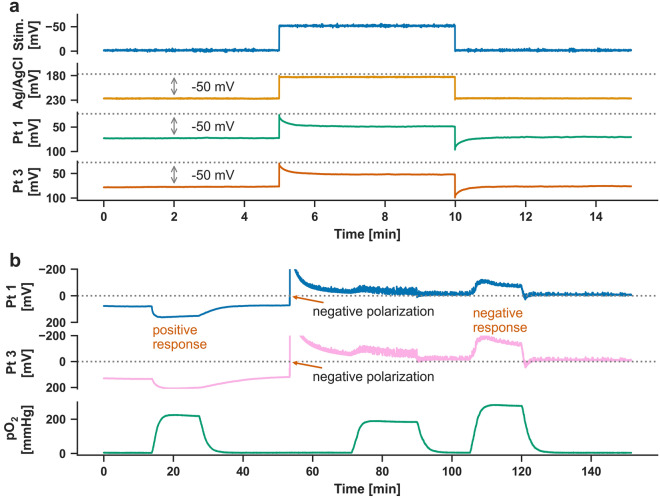
Electrode polarization and effect of positive and negative polarization of Pt electrodes on pO_2_-induced DC artifacts. Electrodes are used to transduce changes in electric potentials into electric currents required by wires and electronic instrumentation. Electrodes consist of electric conductors in contact with the ionic fluids. At the interface between electrode and ionic solution (electric double layer) oxidation–reduction reactions need to occur for a charge to be transferred between electrode and solution. For most electrodes, the cations in solution are of the same metal as the electrode material. The metal atoms either give up electrons (oxidation) and go into solution as positively charged ions or, vice versa, the positively charged metal ions in the solution accept electrons (reduction) and the metal is deposited at the electrode. A commonly used electrode of this type is the Ag/AgCl electrode. The Ag/AgCl electrode is characterized by fast kinetics. This means that the redox reactions (either cathodic deposition of the silver ions or anodic dissolution of the silver metal) are very fast, so the potential of the electrode will not change significantly from its equilibrium potential even if a large, slowly varying voltage is applied to the electrode. This behavior results in a low resistance allowing a high current to pass through the electrode. Therefore, the Ag/AgCl electrode is ideally suited to measure not only changes in AC potential but also DC potential. Electrodes with this behavior in electrical resistance are called nonpolarizable or reversible. Accordingly, electrodes with the opposite behavior are called polarizable. Polarization means that the electrode potential will change significantly from its equilibrium potential with the application of even a small current density because the redox reactions are inherently too slow. The temporary disturbance of the electric double layer at such electrodes by the externally applied voltage leads to only a very small current flow after the first surge, thus indicating a high resistance. This means that the electrode acts as a capacitor: DC is blocked while AC is allowed. Hence, an ideally polarizable electrode will only permit the measurement of AC but not the measurement of DC potentials. However, ideally polarizable electrodes without charge transfer across the interface do not occur in the real world. So-called easily polarizable electrode materials are rather found such as Pt. These can be used to measure DC potentials, but they do so with more or less distortion from the true signal. The nature of this distortion is that of an overpotential, usually calculated as the difference in the electrode potential between the electrode’s equilibrium potential and its operating potential when a current is flowing. As explained above, the electrode’s inherent speed determines the value of the overpotential: the slower the redox reactions, the larger the overpotential for a given current density. **a** DC pulse response recorded on Pt and Ag/AgCl electrodes at pH at 7.4 and pO_2_ at 0 mmHg. In contrast to the Ag/AgCl electrode, the Pt electrode shows a clear distortion from the true signal. Under anoxic conditions, the DC amplitude indicated by the Pt electrode after equilibration was barely half the true DC field potential change given by the external voltage source. In contrast, the Ag/AgCl electrode displayed the true DC shift almost perfectly. Note that negative voltage changes are shown here as upward deflections following the EEG polarity convention. **b** The deflection of the Pt electrode-measured pO_2_-induced DC artifact in the negative or positive direction depended on the negative or positive electrode polarization, respectively, before the pO_2_ increase. An increase in pO_2_ increased the absolute magnitude of the voltage change measured on the Pt electrodes while preserving the polarity so that the pO_2_ increase induced either a positive response starting from a positive baseline or a negative response starting from a negative baseline. AC, alternating current, Ag/AgCl, silver/silver chloride, DC, direct current, EEG, electroencephalography, Pt, platinum

After all electrodes and probes were placed, the pH in the recording chamber was set to 7.4 and pO_2_ to a baseline of 50 mmHg. The electrodes were allowed to stabilize until the DC drift of the Pt electrodes was less than 0.4 mV/min. The effect of changes in pO_2_ was then tested in subsequent steps from 50 mmHg to (1) 0 mmHg, (2) 50 mmHg, (3) 150 mmHg, and (4) 50 mmHg. The same sequence of changes in pO_2_ was then repeated during a pH of 6.4 and during a pH of 8.4. After each environmental change, electrodes were given sufficient time to stabilize. During each pO_2_/pH combination, a square voltage pulse with an amplitude of − 50 mV and duration of 5 min was applied via the stimulation electrode. Only the plateau phase of the voltage curve was used for reading the square pulse amplitude (Fig. 3a).

## Results

### Human recordings

Hyperoxygenation episodes by increasing FiO_2_ at the ventilator started from a p_ti_O_2_ baseline of 37 (30–40) mmHg (median, interquartile range) and reached 71 (50–97) mmHg (Fig. 1a).

During a total of 20 episodes on each of six subdural platinum electrodes in six patients, we observed 95 predominantly negative responses in six patients and 25 predominantly positive responses in four patients. During the same episode, adjacent electrodes could simultaneously show positive and negative responses (Figs. 1a, 2a). The median amplitude of the 95 negative DC responses was 6.2 (4.5–10.4) mV and the amplitude of the 25 positive responses was 7.6 (4.4–10.4) mV. Importantly, the amplifier did not allow us to determine the true baseline voltage of the respective DC recordings. The automatic offset correction alone, which the amplifier performs separately for each channel, thwarts this. Furthermore, a biological electrical voltage source is known to be located between the subdermal and subdural compartments [45–49], and not only the subdural Pt electrodes but also the subdermal Pt quasi-reference is exposed to different chemical adsorbents, pH changes, and pO_2_ changes. Thus, it remained unclear in these measurements whether individual subdural Pt electrodes were negatively polarized, positively polarized, or nonpolarized before the onset of artificial hyperoxygenation episodes.

To highlight the different nature between pO_2_-induced DC artifacts at the Pt electrodes and SDs, we show an SD inducing spreading hyperoxia (Fig. 1b) and another SD inducing spreading hypoxia (Fig. 1c) [14, 43, 44, 50]. These SD-induced O_2_-responses were measured with two intracortical O_2_ sensors at a distance of approximately 1 cm from each other. In contrast to SD-induced spreading hyperoxia and SD-induced spreading hypoxia in the tissue, the artificial hyperoxygenation episode in Fig. 1a was characterized by the simultaneous occurrence of the p_ti_O_2_ increase at both intraparenchymal O_2_ sensors. Figure 2b provides further criteria for how to distinguish pO_2_-induced DC artifacts from SDs. Thus, SD in electrically active tissue leads to a spreading depression of spontaneous activity. This is observed in the AC-ECoG recordings as a rapidly developing reduction in the amplitudes of spontaneous activity that spreads together with SD between adjacent recording sites [36]. In contrast, we did not find significant changes in spontaneous activity during the artificial hyperoxygenation episodes. Furthermore, SD is known to induce tone alterations in resistance vessels, causing either transient hyperperfusion (physiological hemodynamic response) in healthy tissue or initial hypoperfusion (inverse hemodynamic response = spreading ischemia) in tissue where the neurovascular unit is disturbed [12, 24, 51]. Normal and inverse hemodynamic responses to SD typically occur along a continuum in the tissue [1]. The SD in Fig. 2b shows a slightly inverse hemodynamic response at all three optodes. In contrast, we found no significant hemodynamic changes during the artificial hyperoxygenation episodes (Fig. 2a). Table 2 provides an overview of the differences between pO_2_-induced DC artifacts and SDs.

**Table 2:** Comparison of pO_2_-induced DC artifacts and SD

Feature	Systemic-hyperoxia-induced DC artifact	Spreading depolarization
Polarity of DC shift in full-band subdural recordings to a theoretical limit of 0 Hz	Negative or positive	The steep, initial main component is always predominantly negative
Spread of DC shift	Never	Usually, clear spread between adjacent channels
Occurrence in adjacent channels	Occurs in all channels	Some electrodes may not be reached by the SD and therefore these channels do not participate in the DC shift
Duration of DC changes	Depends on the intensivist/nurse that increases the fraction of inspired O_2_	The negative DC shift can last from approximately 50 s [14, 53, 54] to infinity depending on baseline regional cerebral blood flow, baseline tissue partial pressure of O_2_, cerebral metabolic rate of O_2_ and the hemodynamic response to SD, an infinite negative DC shift can occur, for example, during the development of a cerebral infarction or during dying [12, 17–19]
Depression of spontaneous brain activity	No depression	Spreading depression if spontaneous activity was present at onset of SD and AC-ECoG recordings are of sufficient quality; spreading depression is observed in AC-ECoG recordings as a rapidly developing reduction in the amplitudes of spontaneous activity
Regional cerebral blood flow response	No significant change	Either spreading hyperemia (normal response) or spreading ischemia (inverse response) or no change
O_2_ response if recorded with O_2_ sensors at different locations	Simultaneous increase at adjacent O_2_ sensors	Either spreading hyperoxia or spreading hypoxia or no change

*AC* alternating current, *DC* direct current, *ECoG* electrocorticography, *SD* spreading depolarization

### Changes in pO_2_ and pH result in DC shifts at Pt electrodes

The simple placement of the Cu electrode for stimulation in the in vitro recording chamber induced a DC change in the positive direction at the Pt electrodes. For this, no external current had to be applied. Increases in pO_2_ from the baseline, then, always led to additional positive DC shifts under this condition of a positive electrode polarization (Fig. 3b). External application of a constant negative voltage with an amplitude of 1 V to the stimulation electrodes subsequently led to a persistent negative DC change at the Pt electrodes and an increase in noise that was most intense around 0.5 Hz. Under this condition of a negative electrode polarization, increases in pO_2_ from baseline always led to additional negative DC shifts (Fig. 3b).

Accordingly, this experiment suggested that the hyperoxygenation-induced DC artifacts at the Pt electrodes could depend on the direction in which the respective electrodes are polarized under baseline conditions.

**Fig. 4:**
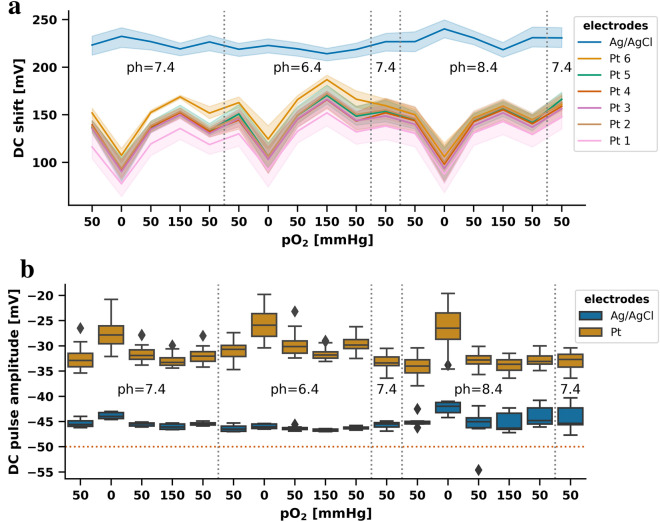
Effects of pO_2_ and pH changes on the DC potential, and DC pulse responses under different conditions of pO_2_ and pH. **a** Changes in pO_2_ and pH markedly shifted the DC voltage at the Pt electrodes in a nonlinear fashion. The DC shift amplitudes due to pO_2_ changes did not differ between different pH values, but the shift in pH itself also induced DC shifts. The lines represent the mean and the shading the 95% confidence interval of the measured values for each electrode across all experiments. From left to right, each line starts at the baseline value which was measured at a pO_2_ of 50 mmHg and a pH of 7.4, and the voltage changes from one condition to the next condition are shown. The polarity of the shifts in relation to the preceding condition is given by the *y*-axis of the graph. Note that positive voltage changes are shown in this more technical figure as upward deflections. Values were determined after a given pO_2_ or pH change, when the electrodes had stabilized and before the square voltage pulse was applied. **b** During the different levels of pO_2_ and pH, we applied 5 min-long, negative square voltage pulses with an amplitude of 50 mV to test whether the effect of electrode polarization varies at different pO_2_ and pH levels. The amplitude of the square − 50 mV pulse (marked with a red horizontal line) was never perfectly reproduced by either Ag/AgCl or Pt electrodes but the measurements with the Ag/AgCl electrode were always superior to the measurements with the Pt electrodes. The Pt electrodes showed the poorest performance under anoxic conditions. Ag/AgCl, silver/silver chloride, DC, direct current, Pt, platinum

In six other experiments, we investigated only the low-noise condition of positive electrode polarization without external current application as an example. Under this condition, we determined the DC responses to pO_2_ and pH changes in the recording chamber. The Pt electrodes showed a baseline of 136.9 (131.1–147.0) mV at the onset of the experiments when pH was 7.4 and pO_2_ was at 50 mmHg (Fig. 4a). Decrease in pO_2_ to 0 mmHg resulted in a negative DC shift with an amplitude of 38.5 (36.2–51.2) mV and return to 50 mmHg in a positive DC shift with an amplitude of 42.4 (34.6–48.2) mV. A further increase to 150 mmHg led to a positive DC shift with an amplitude of only 15.9 (15.3–16.6) mV, indicating that the effect of pO_2_ on the DC potential was nonlinear. Acidification from a pH of 7.4 to 6.4 led to a positive DC shift of 13.5 (7.7–19.9) mV and alkalinization from a pH of 7.4 to 8.4 to a negative DC shift of 7.3 (11.6–5.4) mV. The pO_2_-induced DC shifts were similar under pH 6.4, 7.4 or 8.4. The Ag/AgCl electrode was also influenced by the environmental changes but to a much lesser extent than the Pt electrodes (Fig. 4a).

### Influence of pO_2_ and pH on the Pt recorded square voltage pulses

In the six experiments, we additionally investigated whether the pO_2_ changes and pH changes modified DC field potential changes in response to square voltage pulses with an amplitude of − 50 mV and a duration of 5 min. In response to a square voltage pulse of − 50 mV, the Ag/AgCl electrode recorded a DC shift of 45.5 (45.2–45.9) mV at a pH of 7.4 and a pO_2_ of 50 mmHg. During all investigated combinations of pO_2_ in neutral and acidic pH, the amplitude of the recorded negative DC shift was in the range between 43.0 and 47.0 mV (Fig. 4b). The Ag/AgCl electrode showed worse performance in alkaline than in neutral or acidic medium increasing the measurement range to 33.8–54.6 mV. Pt electrodes performed always worse than the Ag/AgCl electrode. Their poorest performance was under anoxic conditions, where they indicated little more than 50% of the true negative DC field potential shift (example in Fig. 3a) and showed the largest variability (Fig. 4b). Median and interquartile range of the amplitudes measured on Pt and Ag/AgCl electrodes during 50 mV square voltage pulses under different pO_2_ and pH values are given in Table 3.

**Table 3:** Amplitudes measured on Pt and Ag/AgCl electrodes during 50-mV square voltage pulses under different pO_2_ and pH values

pH	7.4					
pO_2_ (mmHg)	50	0	50	150	50	
Ag/AgCl (mV) (*n* = 6)	46 (45–46)	44 (43–44)	45 (45–46)	46 (46–47)	46 (45–46)	
Pt (mV) (*n* = 36)	33 (32–34)	28 (26–30)	32 (31–33)	33 (32–34)	32 (31–33)	

pH	6.4					7.4
pO_2_ (mmHg)	50	0	50	150	50	50
Ag/AgCl (*n* = 6) (mV)	46 (46–47)	46 (46–46)	46 (46–47)	47 (47–47)	46 (46–46)	46 (45–46)
Pt (*n* = 36) (mV)	31 (30–32)	26 (24–28)	30 (29–31)	32 (31–32)	30 (29–31)	33 (32–34)

pH	8.4					7.4
pO_2_ (mmHg)	50	0	50	150	50	50
Ag/AgCl (*n* = 6) (mV)	45 (45–45)	42 (41–43)	45 (44–46)	46 (43–47)	45 (42–46)	45 (42–46)
Pt (*n* = 36) (mV)	34 (33–35)	27 (23–29)	33 (32–34)	34 (33–35)	33 (32–34)	33 (32–34)

An ideal electrode would measure 50 mV. Ag/AgCl electrodes also failed to reach 50 mV, but Pt electrodes were clearly worse. Data are presented as medians and interquartile ranges

*Ag*/*AgCl* silver/silver chloride, *DC* direct current, *Pt* platinum

## Discussion

Transient worsening of mismatch between oxidative substrate supply and demand was a potent stimulus for delayed SDs in a previous study in mice after middle cerebral artery occlusion [42]. Such transient worsening could result not only from systemic arterial blood pressure fluctuations but also, for example, from sensory stimulation of metabolically unstable cortical representation fields. It is therefore likely that manipulations on patients during neurocritical care may lead to SDs in some circumstances. Potentially, SDs triggered in this way could contribute to lesion growth [4, 5, 52]. Therefore, in neuromonitored patients in the intensive care unit, it is recommended to pay attention to whether SDs occur during manipulations [42]. On the other hand, patients are typically subjected to brief episodes of hyperoxygenation before manipulations are performed on them, especially manipulations around the endotracheal tube. In the present work, we have investigated pO_2_-induced DC shifts that result as electrode artifacts from such hyperoxygenation episodes. They should not be confused with SDs.

The magnitude of the pO_2_-induced subdural DC artifacts by approximately 6 mV is exactly in the same range as DC shifts from SDs, but DC shifts of SDs typically show a sequential onset at adjacent recording sites and their steep, initial component is always predominantly negative in full-band subdural recordings [14, 22, 53, 54]. In contrast, pO_2_-induced DC artifacts started simultaneously at all electrodes and, surprisingly, they could be negative and positive at adjacent electrodes at the same time. Other differences between pO_2_-induced electrode artifacts and SDs are summarized in Table 2.

We found that the change in polarity of the pO_2_-induced DC artifacts in our in vitro experiments could be produced by reversing the polarization of a Pt cathode (Fig. 3b). When no voltage was applied, the arrangement of Pt cathode and Cu anode constitutes a galvanic cell in which the Pt cathode is positively charged according to the positions of Pt and Cu in the electrochemical series. When the Pt cathode was negatively polarized by a constant voltage of approximately 1 V, the galvanic cell was turned into a so-called electrolytic cell, in which redox reactions take place that normally do not occur voluntarily, i.e., chemical reactions are forced to take place in the opposite direction instead of in their normally energetically favorable direction. Through the universal concept of energy, these paradoxical redox reactions, like normal redox reactions, are associated with electrical voltage changes that can be calculated using the Nernst equation. If chemical reactions are forced in an electrolytic cell, which are accompanied by voltage changes and proceed in the opposite direction to the galvanic cell, it may be hypothesized that not only the direction of the chemical reactions but also the polarity of the voltage changes is reversed in the electrolytic compared to the galvanic cell. This could be a basic explanation for the polarization-dependent change in polarity of the pO_2_-induced DC artifacts.

The exact description of the underlying redox reactions is however beyond the scope of this clinical paper as the electrochemical reaction of O_2_ at a Pt surface is surprisingly complex, even in an aqueous electrolyte solution without additional chemical species. This so-called oxygen reduction reaction at the Pt surface is in fact one of the best studied chemical reactions, but its details are still considered not understood in chemistry today. In the simple version, O_2_ is spontaneously reduced to water in a 4-electron process via at least two different routes (O_2_ + 4 e^−^  + 4 H^+^  ⇆ 2 H_2_O or O_2_ + 2 e^−^  + 2 H^+^  ⇆ 2 H_2_O_2_) at near neutral pH [55]. The kinetics of this reaction depend on the solution pH, electrolytes, pretreatment of the Pt surface, pO_2,_ and the electrode polarization [55–57]. Under atmospheric pressure, at nearly neutral pH and low or negatively polarized Pt cathode, chemisorbed hydroxyl ions are formed as the dominant intermediate surface oxide during O_2_ adsorption and reduction, as was suggested by several authors [56–58]. It is assumed that the latter is the decisive redox reaction via which pO_2_ is measured in so-called Clark-type electrodes, which consist of an electrolytic cell with Pt cathode and Ag anode. For this reaction to proceed efficiently in a physiological electrolyte at near neutral pH and ambient pressure, a substantial negative polarization of approximately 200 mV or more versus the standard hydrogen electrode is required. It could be argued that strong negative polarization, which would be sufficient for this response, is not expected per se at the subdural Pt electrodes in patients in situ because the electrodes are not connected to any obvious current source. However, it is well known that transient surface oxides can easily form at Pt surfaces under normal atmospheric conditions exhibiting a surprising variety of structural patterns [56, 57]. The actual electrochemical processes at the surface of the Pt electrodes in patients in situ will be even more complex than in test tubes due to adsorbed molecules such as, for example, various hemoglobin species containing either Fe^2+^ or Fe^3+^ ions. Hemoglobin molecules could get into contact with the Pt electrodes in situ because the electrodes were implanted in patients after aSAH. In addition to hemoglobins, other proteins, small aromatic molecules, and/or thiol compounds could be adsorbed at the Pt surface, further modifying the features of the electrodes. Therefore, even a passively recording Pt electrode may become partially polarized, e.g., due to local currents [38], and the polarization between adjacent electrodes could differ due to locally different concentrations of adsorbed molecules. Accordingly, different polarities of pO_2_-induced DC artifacts would result. The Cu electrode is a rather artificial addition generating chemical current in our in vitro setup. However, we consider the Cu electrode as a placeholder of biological current sources known to exist in vivo. For example, the cortical surface of the physiological mammalian brain is steadily positive to other parts of the nervous system such as the cerebral ventricles by approximately 10–20 mV [45–49].

However, there could also be an alternative more trivial explanation for the fact that the pO_2_-induced DC artifacts at adjacent electrodes can be negative and positive at the same time, which is related to the subdermal quasi-reference. A “true” reference electrode is an electrode with a thermodynamically precisely defined redox potential, i.e., its potential can be traced back to the potential of the standard hydrogen electrode. As usual in electrochemistry, the term “electrode” implies the electrode material and the electrolyte solution in which it is immersed. The calomel electrode (Hg/HgCl) or the Ag/AgCl electrode are examples of true reference electrodes whose potentials theoretically depend only on the chloride ion concentration of the surrounding electrolyte solution. In contrast, a metal (e.g., Pt) or metal oxide electrode (e.g., iridium dioxide) is a “quasi-reference” electrode. The potential of a quasi-reference is much less precisely defined than the potential of a true reference as it depends in a complex fashion on the electrolyte in which it is immersed. Thus, the potential of the Pt quasi-reference in the patients is floating depending on pO_2_, pH, anion and cation species present, chemisorbing molecular species, etc., in the subdermal compartment. In other words, the potential of the subdermal Pt quasi-reference will change during a systemic hyperoxygenation episode as will the potential of the subdural Pt electrodes. In principle, the potential of all electrodes could change in the same direction, but by a slightly different amount in each case due to local inhomogeneities of the pO_2_ changes in the different compartments. Depending on whether the local pO_2_ change at a given subdural electrode is larger or smaller than the local pO_2_ change at the subdermal quasi-reference, the measured potential change could then be either negative or positive.

In addition to pO_2_-induced DC artifacts, we also investigated pH-induced DC artifacts of the Pt electrodes in our in vitro setup as extracellular pH changes are well known to occur in patients. For example, acidosis typically develops during brain ischemia [59, 60]. The pH-induced DC artifacts were somewhat less dramatic than the pO_2_-induced DC artifacts, but they were still not negligible. The pH-induced DC artifacts occur because Pt also adsorbs H^+^ ions upon electron release (H_ads_ ⇆ H^+^  + e^−^), which then may be released as H_2_ (H_2_ ⇆ 2 H_ads_)  [61].

Another interesting finding was that the lower the pO_2_, the less accurately the square voltage pulses were recorded by the Pt electrodes. Polarizable electrodes behave like a parallel-connected resistor and capacitor [38], with capacitance dominating the electrode behavior [37, 38]. Although they reproduce high-frequency signals nearly perfectly, the amplitude of signals in the low-frequency DC range is distorted inversely proportional to the frequency. Depending on the electrical circuit, Pt electrodes may even return to the baseline during a square pulse stimulus [38]. Overall, our results further support the notion that the combined effects of O_2_ adsorption, surface roughening, and catalytic reactions at the Pt surface improve the electrode properties. This may be the reason why long-term recording in patients with subdural Pt electrodes is at all possible [40].

Limitations of our study include the relatively small number of patients and events, the retrospective design, and the fact that it was not precisely documented why intensivists and nurses had each increased FiO_2_. However, we would like to reiterate that brief episodes of hyperoxygenation before performing patient manipulations are a standard procedure routinely done in intensive care units around the world in a similar fashion.

## Conclusions

If neurointensivists wish to use Pt electrodes to predict and detect ischemic conditions in the brain by monitoring SDs, the awareness of pO_2_ and pH interference with Pt electrodes is important because changes in pO_2_ and pH can produce large DC artifacts that should not be confused with SDs. Second, automatic algorithms for SD detection should be developed in such a way to reliably distinguish pO_2_-induced DC artifacts from SDs. Third, these interferences are one of the many reasons why it is more meaningful to perform monitoring of SDs with subdural Pt electrodes outside the brain parenchyma than with Pt electrodes located directly in the parenchyma [36, 40]. SDs cause marked p_ti_O_2_ changes in brain tissue during which p_ti_O_2_ can either increase or decrease, depending on baseline cerebral blood flow, baseline p_ti_O_2_, cerebral metabolic rate of O_2_, and hemodynamic responses to SDs [14, 43, 44, 50] (Figs. 1b, c, 2b). Moreover, SDs cause marked tissue acidosis [59, 60]. If the Pt electrodes are located directly in the brain parenchyma [62], the DC signal consists not only of the field potential of SD but also of interference artifacts from p_ti_O_2_ and tissue pH with the Pt electrodes, which greatly complicates interpretation. Hartings and colleagues previously found that monitoring only a single cortical location, as with an intraparenchymal depth array, may fail to capture 43% of SDs that occur in a broader area of subdural strip monitoring [40]. This already inadequate detection rate may be further worsened by signal cancellation if the Pt electrode is located directly in the brain parenchyma instead of subdurally and the negative DC shift of the SD is superimposed, for example, by a positive DC shift resulting from the SD-induced p_ti_O_2_ response in the parenchyma and O_2_ interference with the Pt electrode. In contrast, if the Pt electrodes are located in the subdural space, the DC signal consists mainly of the field potential of SD because O_2_ tension and pH of the subdural space depends on perfusion of the capillaries of the meninges and not the capillaries of the brain. As SD is a gray matter phenomenon and not a meningeal phenomenon, the changes in meningeal blood flow, if any, are minor compared with the changes in cortical blood flow during SD. This means that the subdural space is generally a much more electrochemically stable compartment than the brain parenchyma. In addition, histological and immunohistochemical analyses of brain tissue surgically resected from patients with epilepsy suggested that depth electrodes cause upregulation of active inflammatory cell types and extravasation of plasma proteins, indicating significant local disruption of the blood–brain barrier, in an area that is 30 times the area of the physical insult [63]. Because the field potential changes of SDs in the subdural compartment are still large enough to be unambiguous [12, 14, 22, 23, 53, 54], in contrast with those in the epidural space or at the scalp surface [18, 64, 65], our results provide further evidence that the subdural compartment is the ideal location for measuring SDs with Pt electrodes. Nevertheless, there are also arguments for the use of depth electrodes under certain circumstances. For a more comprehensive account of subdural versus depth electrodes we would like to refer to the COSBID recommendations on recording, analysis, and interpretation of SDs in neurointensive care [36]. Overall, the development of materials that are better than Pt should continue so that mechanically robust, nontoxic, nonpolarizable, noncatalytic electrodes with low impedance will be available for monitoring SDs in the future [66, 67].

## Supplementary Information

Below is the link to the electronic supplementary material.

Supplemental Fig. 1: In vitro setup. The gas inflow (N_2_/O_2_) was placed in the recording chamber together with a 6-contact platinum (Pt) electrode strip, a single Ag/AgCl electrode and a stimulation electrode connected to a power source. In addition, a pH sensor and an O_2_ sensor (Pico-ammeter PA 2000, Unisense, Aarhus, Denmark) were placed in the recording chamber. The artificial cerebrospinal fluid (ACSF) in the recording chamber was continuously bubbled with a mixture of N_2_ and O_2_. Changes in the partial pressure of O_2_ (pO_2_) were achieved by changing the proportion between N_2_ and O_2_. Changes in pH were achieved by addition of either NaOH or HCl and verified by the pH meter. In order to generate square voltage pulses (5min duration), copper electrodes connected to an adjustable power source were placed in each chamber. After electrode stabilization, the experiments started at physiological levels of pH and pO_2_. Subsequently, episodes of hypoxia/hyperoxia and acidosis/alcalosis were induced. The DC potential was always permitted to reach steady-state. During the different levels of pO_2_ and pH, we applied 5min-long, negative square voltage pulses to test whether the effect of electrode polarization varies at different pO_2_ and pH levels. ADC: analog-digital converter, Amp: differential amplifier (Jens Meyer, Munich, Germany), sb: salt bridge.
