# Supplementary figures and images for: Oxygen-Induced and pH-Induced Direct Current Artifacts on Invasive Platinum/Iridium Electrodes for Electrocorticography
Source: Neurocrit Care. 2021 Oct 7;35(Suppl 2):146–59. doi: 10.1007/s12028-021-01358-2 (PMC8496677; doi:10.1007/s12028-021-01358-2)

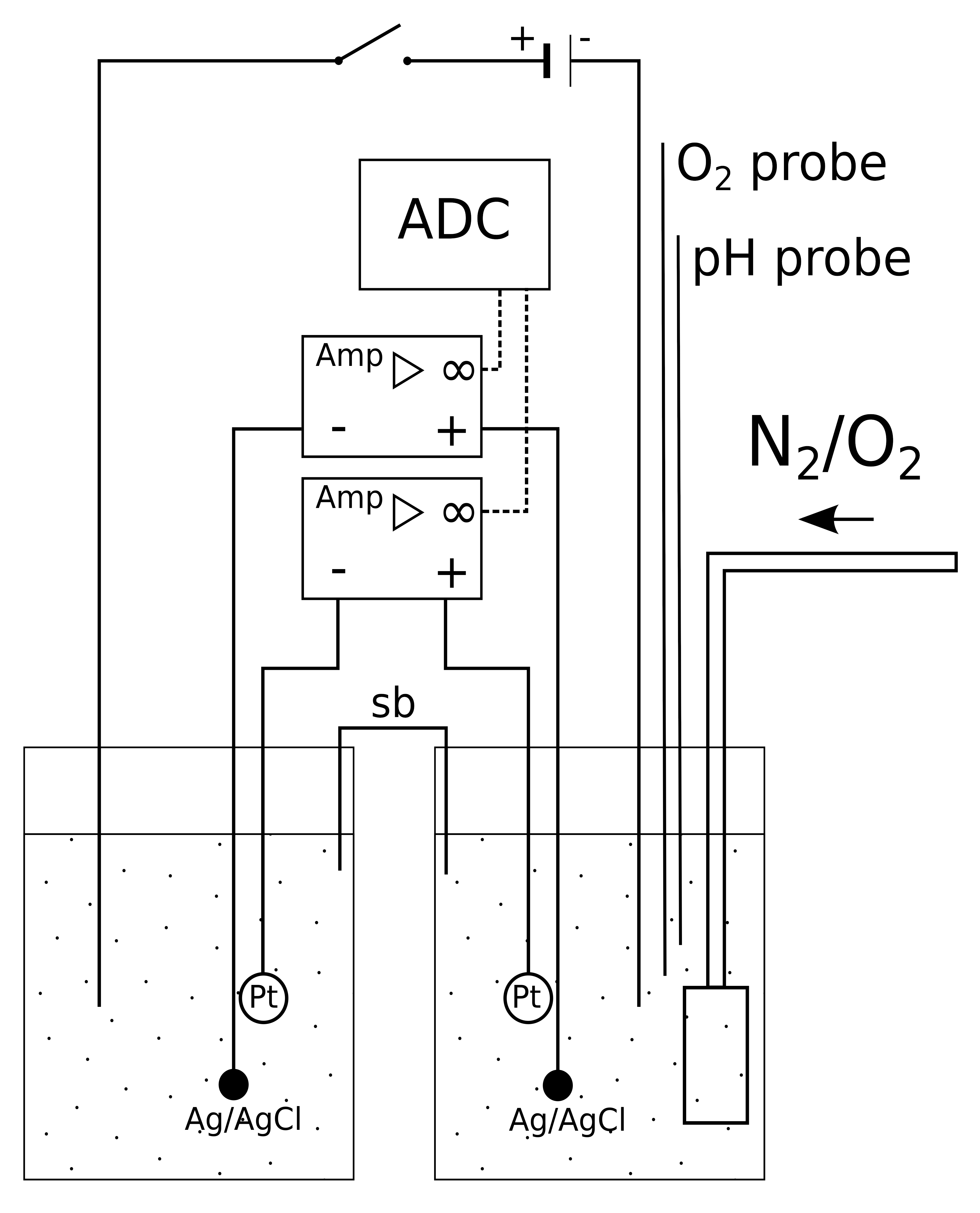

Supplement: Supplementary file 1 — Supplemental Fig. 1: In vitro setup. The gas inflow (N2/O2) was placed in the recording chamber together with a 6-contact platinum (Pt) electrode strip, a single Ag/AgCl electrode and a stimulation electrode connected to a power source. In addition, a pH sensor and an O2 sensor (Pico-ammeter PA 2000, Unisense, Aarhus, Denmark) were placed in the recording chamber. The artificial cerebrospinal fluid (ACSF) in the recording chamber was continuously bubbled with a mixture of N2 and O2. Changes in the partial pressure of O2 (pO2) were achieved by changing the proportion between N2 and O2. Changes in pH were achieved by addition of either NaOH or HCl and verified by the pH meter. In order to generate square voltage pulses (5min duration), copper electrodes connected to an adjustable power source were placed in each chamber. After electrode stabilization, the experiments started at physiological levels of pH and pO2. Subsequently, episodes of hypoxia/hyperoxia and acidosis/alcalosis were induced. The DC potential was always permitted to reach steady-state. During the different levels of pO2 and pH, we applied 5min-long, negative square voltage pulses to test whether the effect of electrode polarization varies at different pO2 and pH levels. ADC: analog-digital converter, Amp: differential amplifier (Jens Meyer, Munich, Germany), sb: salt bridge. [file 12028_2021_1358_MOESM1_ESM.tif]
